# Flightless-1 inhibits ER stress-induced apoptosis in colorectal cancer cells by regulating Ca^2+^ homeostasis

**DOI:** 10.1038/s12276-020-0448-3

**Published:** 2020-06-05

**Authors:** Sun Sil Choi, Sang Kwon Lee, Joong Kwan Kim, Hye-Kyung Park, Eujin Lee, Jinho Jang, Yo Han Lee, Keon Woo Khim, Ji-Min Hyun, Hye-jin Eom, Semin Lee, Byuong Heon Kang, Young Chan Chae, Kyungjae Myung, Seung-Jae Myung, Chan Young Park, Jang Hyun Choi

**Affiliations:** 1 Department of Biological Sciences, Ulsan National Institute of Science and Technology (UNIST), Ulsan, 689-798 Korea; 2 Department of Biological Sciences, Center for Genomic Integrity (CGI), Institute for Basic Science (IBS), Ulsan National Institute of Science and Technology (UNIST), Ulsan, 689-798 Korea; 3 Department of Gastroenterology, University of Ulsan College of Medicine, Asan Medical Center, Seoul, 05505 Korea

**Keywords:** Apoptosis, Cancer

## Abstract

The endoplasmic reticulum (ER) stress response is an adaptive mechanism that is activated upon disruption of ER homeostasis and protects the cells against certain harmful environmental stimuli. However, critical and prolonged cell stress triggers cell death. In this study, we demonstrate that Flightless-1 (FliI) regulates ER stress-induced apoptosis in colon cancer cells by modulating Ca^2+^ homeostasis. FliI was highly expressed in both colon cell lines and colorectal cancer mouse models. In a mouse xenograft model using CT26 mouse colorectal cancer cells, tumor formation was slowed due to elevated levels of apoptosis in FliI-knockdown (FliI-KD) cells. FliI-KD cells treated with ER stress inducers, thapsigargin (TG), and tunicamycin exhibited activation of the unfolded protein response (UPR) and induction of UPR-related gene expression, which eventually triggered apoptosis. FliI-KD increased the intracellular Ca^2+^ concentration, and this upregulation was caused by accelerated ER-to-cytosolic efflux of Ca^2+^. The increase in intracellular Ca^2+^ concentration was significantly blocked by dantrolene and tetracaine, inhibitors of ryanodine receptors (RyRs). Dantrolene inhibited TG-induced ER stress and decreased the rate of apoptosis in FliI-KD CT26 cells. Finally, we found that knockdown of FliI decreased the levels of sorcin and ER Ca^2+^ and that TG-induced ER stress was recovered by overexpression of sorcin in FliI-KD cells. Taken together, these results suggest that FliI regulates sorcin expression, which modulates Ca^2+^ homeostasis in the ER through RyRs. Our findings reveal a novel mechanism by which FliI influences Ca^2+^ homeostasis and cell survival during ER stress.

## Cancer: Protein enhances survival of tumor cells under stress

A cytoskeletal protein that helps tumors avoid cell death offers a promising new drug target for fighting cancer. A team led by Jang Hyun Choi and Sun Sil Choi of the Ulsan National Institute of Science and Technology, South Korea, detailed how a protein called Flightless I (FliI) that normally regulates the remodeling of structural filaments in the cell can, in colorectal cancer cells, serve as a tumor promoter through its action on calcium levels. Typically, cells respond to chronic stress by altering calcium signaling to promote their own death. In tumors, however, FliI maintains normal calcium levels to enhance cell survival even in the face of chemotherapy and other stressful stimuli. Suppressing FliI activity could thus help sensitize cancer cells to other stress- and death-inducing drug regimens.

## Introduction

Colorectal cancer (CRC) is one of the most common lethal cancers around the world^1^. The disease is characterized by local recurrence, distant metastasis, and extremely rapid progression^1^. Surgical resection, systemic chemotherapy, and radioembolism are the major strategies for treating CRC, but high mortality and poor prognosis remain serious clinical problems^1^. The main objective of cancer therapy is to destroy all cancer cells while causing minimal damage to normal tissue. Apoptosis, a genetically regulated form of programmed cell death, is an emerging target for anticancer therapy^2^. In general, the activation of apoptosis is an important mechanism for the prevention and treatment of CRC, as well as other cancers^2^. Three different pathways lead to apoptosis: the extrinsic death receptor pathway, the intrinsic mitochondrial pathway, and the endoplasmic reticulum (ER) stress pathway^2^. The mechanisms involved in the intrinsic and ER stress-mediated pathways influence each other. Accordingly, the ER stress pathway represents an emerging therapeutic target for cancer treatment^3^.

The ER is a multifaceted organelle involved in initial protein maturation, lipid synthesis, and maintenance of intracellular calcium homeostasis^4^. Disruption of ER homeostasis following glucose deprivation, hypoxia, disruption of Ca^2+^ homeostasis, or accumulation of misfolded proteins activates the unfolded protein response (UPR) and initiates an intracellular signaling pathway that protects the cell^4^. Under normal conditions, three ER stress sensors, protein kinase-like ER kinase (PERK), activating transcription factor 6 (ATF6), and inositol-requiring kinase-1 (IRE1), are bound to the ER-resident chaperone glucose-regulated protein-78 (GRP78/BiP)^5^. However, under ER stress, these proteins are released from GRP78/BiP and initiate their downstream cascades. IRE1α initiates the splicing of X-box binding protein-1 (XBP-1) mRNA. Spliced XBP-1 functions as a transcriptional activator of UPR target genes, including GRP78/BiP and calreticulin^5^. PERK is a transmembrane kinase that phosphorylates and inactivates eukaryotic translation initiation factor 2 subunit α, leading to reduced protein synthesis^5^. The PERK/EIF1α pathway also activates ATF4, which upregulates CCAAT/enhancer-binding protein-homologous protein (CHOP). Concomitantly, during ER stress, cleaved and activated ATF6α translocates to the nucleus and transactivates the genes encoding various chaperones and ER stress markers, including CHOP and GRP78/BiP itself^5^. Activation of the UPR promotes cell survival by decreasing protein synthesis and increasing protein folding capacity. However, if ER stress persists and homeostasis cannot be restored, the UPR triggers cell death in cells that are beyond repair^6^.

Ca^2+^ is a ubiquitous intracellular messenger that controls multiple cellular functions, including transcription, exocytosis, apoptosis, and proliferation. Ca^2+^ is mainly stored in the ER, and its levels are tightly regulated by multiple pumps, channels, and binding proteins in that organelle^7^. Ca^2+^ movement across the ER membrane is mediated by Ca^2+^ release channels, ryanodine receptors (RyRs), ER-resident inositol 1,4,5-trisphosphate receptor (IP_3_Rs), and the Ca^2+^ uptake pump sarco-ER Ca^2+^-ATPase (SERCA)^8–10^. However, despite tight regulation of ER Ca^2+^, alterations in ER Ca^2+^ homeostasis due to dysfunction of these proteins or critical and continuous ER stress provoke ER Ca^2+^ depletion and an overload of intracellular Ca^2+^, resulting in excessive Ca^2+^ accumulation in the mitochondria. This, in turn, causes apoptosis by increasing mitochondrial membrane permeabilization and promoting the release of cytochrome *c*^11^.

Flightless-1 (FliI), originally identified as a *Drosophila melanogaster* mutant, is a member of the gelsolin superfamily with an N-terminal leucine-rich repeat domain and a C-terminal gelsolin-like domain^12^. Through its bipartite domain structure, FliI can bind to numerous structural and signaling proteins and thus regulate cell migration, wound healing, and inflammation^13–17^. The main roles of the gelsolin family are Ca^2+^- and phosphatidylinositol 4,5-bisphosphate-regulated actin binding^18^. However, FliI is more divergent from gelsolin than other family members, and its actin-binding and actin-severing activities are Ca^2+^ independent^19^. By contrast, FliI interacts in a Ca^2+^-dependent manner with nonmuscle myosin IIA, which plays an essential role in cell extension by activating transient receptor potential cation channel subfamily V member 4^20^. Furthermore, FliI modulates cell proliferation and survival in cancer cells by interacting with transcription factors such as androgen receptor, estrogen receptor (ER), and carbohydrate responsive element-binding protein, which regulate tumor progression in prostate cancer and CRC cells^21–23^. Recently, FliI was shown to promote breast cancer progression by impeding selective autophagy through an interaction with p62^24^. Here, we report a novel function of FliI: FliI suppresses ER stress-induced UPR signaling and apoptosis in colon cancer by regulating Ca^2+^ homeostasis through modulation of RyR activity.

## Materials and methods

### Cell culture, stable cell line generation

CT26 (ATCC: CRL-2368) cells were cultured in RPMI 1640 medium supplemented with 10% fetal bovine serum (GIBCO BRL, Grand Island, NY, USA) and antibiotics (100 U/ml penicillin and 100 ug/ml streptomycin). Cells were grown at 37 °C under a humidified 5% CO_2_ atmosphere. The sequence used for the lentiviral shRNA expression vector (pLKO.1; Open Biosystems, Huntsville, AL, USA) targeting FliI was 5′-TTCTAGGTTGTTGTTGGCAGC-3′. For lentivirus production, HEK-293T cells (ATCC; Manassas, VA, USA) were transfected with 10 µg lentiviral vectors. Following infection with lentivirus, cells were selected with 1 μg/ml puromycin.

### Live-cell imaging for intracellular calcium

shRNA-Ctrl or shRNA-FliI CT26 cells were incubated at 37 °C for 30 min in media containing 1 μM Fluo-4AM (Invitrogen, Carlsbad, CA, USA). After washing with Hank’s buffer, cells were analyzed by flow cytometry or imaged on a fluorescence microscope (Olympus, 20×), with excitation and emission wavelengths of 488 and 505 nm, respectively. For analysis using GCaMP6s, shRNA-Ctrl or shRNA-FliI CT26 cells were seeded on a cover glass and transfected with pDEST-mCherry-GCaMP6s using Lipofectamine 2000 (Invitrogen, Carlsbad, CA, USA). After 24 h, intracellular Ca^2+^ was imaged on an IX83 microscope (Olympus) equipped with an Olympus 40× objective lens (oil, NA 1.30), a fluorescent lamp (Olympus), a stage controller (LEP), and a CCD camera (Andor, Concord, MA, USA). Images were processed with MetaMorph software (Molecular Devices, San Jose, CA, USA). For ratiometric Ca^2+^ imaging, cells were pretreated for 2 h with dantrolene (50 µM; Sigma-Aldrich, St. Louis, MO, USA), 2-APB (50 µM; Sigma-Aldrich, St. Louis, MO, USA), tetracaine (50 μM; Sigma-Aldrich), DBHQ (2,5-di-tert-butylhydroquinone; 1 μM; Santa Cruz Biotechnologies, Santa Cruz, CA, USA), and loaded with 1 µM Fura-2AM (Molecular Probes) for 30 min. Ratiometric Ca^2+^ imaging at 340 and 380 nm was performed at room temperature in calcium-free Tyrode’s solution (129 mM NaCl, 5 mM KCl, 3 mM MgCl_2_, 30 mM glucose, and 25 mM HEPES [pH 7.4]) with or without 5 µM ionomycin, 1 μM thapsigargin (TG), and 50 μM DBHQ on an IX81 microscope equipped with an Olympus 40× objective lens (oil, NA 1.30), a fluorescent arc lamp (Lambda LS), an excitation filter wheel (Sutter, Lambda 10–2), a stage controller (ASI, MS-2000), and a CCD camera (Hamamatsu, C10600). Images were processed using MetaMorph software (Molecular Devices) and analyzed using Igor Pro software (WaveMetrics, Portland, OR, USA).

### Analysis of cell viability and apoptosis induction

shRNA-Ctrl or shRNA-FliI CT26 cells (1 × 10^4^ cells/well) were cultured in 96-well plates overnight and then treated with TG for 48 h. To determine cell viability, cells were exposed to 3-(4,5-dimethyl-thiazol-2-yl)2,5 diphenyltetrazolium bromide (MTT; Sigma-Aldrich), and crystallized formazan was quantified by measuring absorbance at 595 nm on an Infinity M200 microplate reader (Tecan, Männedorf, Switzerland). Absorbance data were normalized against the vehicle control and expressed as percent viability. Alternatively, after treatment with TG, shRNA-Ctrl or shRNA-FliI CT26 cells were stained with annexin V-FITC (Invitrogen) and propidium iodide (PI; Invitrogen) and quantified on a FACSCalibur system (BD Biosciences, San Jose, CA, USA). Data were processed using FlowJo software (BD Biosciences). For colony formation assays, shRNA-Ctrl or shRNA-FliI CT26 cells (1 × 10^3^ cells/well) were treated with TG for 48 h, after which the medium was replaced with drug-free medium, and the cells were incubated for 14 days. The colonies were washed with PBS, fixed with methanol, and stained with crystal violet.

### Transient transfection of plasmid DNA

Mouse sorcin expression plasmids were purchased from Sino Biological (BDA, Beijing, China). Plasmid DNA was transiently transfected into FliI-KD CT26 cell lines using jetOptimus (Polyplus-transfection, Illkirch-Graffenstaden, France). Cells were treated with TG at the indicated concentration to induce ER stress.

### ER Ca^2+^ measurement

For ER calcium measurements, cells were transiently transfected with a plasmid encoding the D1ER Cameleon. pcDNA-D1ER was a gift from Amy Palmer & Roger Tsien (Addgene plasmid #36325)^25^. ZEN (Zeiss, Oberkochen, Germany) was used to calculate fluorescence intensities from images taken under three conditions: IDD, donor fluorescence intensity excited by an excitation laser (458 nm); IDA, acceptor intensity under a donor excitation laser; and IAA, acceptor intensity excited by an acceptor excitation laser (514 nm). The FRET efficiency is defined as follows:^26^ FRET efficiency = (*I*_DA_ – *β* × *I*_DD_ − *γ* × *I*_AA_)/*I*_AA_, where *β* is a bleeding-through coefficient from donor to acceptor channels and *γ* is the ratio of fluorescence intensity of acceptor molecules directly excited by a donor excitation laser to fluorescence intensity of acceptor molecules excited by an acceptor excitation laser.

### Immunoblotting

Samples were lysed in RIPA lysis buffer (50 mM Tris, pH 8.0, 150 mM NaCl, 1% NP-40, 0.1% SDS, and 0.25% N-deoxycholate) containing protease and phosphatase inhibitors (Sigma-Aldrich). Equal amounts of protein were separated by SDS-PAGE and transferred onto nitrocellulose membranes (GE Healthcare, MA, USA). Membranes were blocked in 5% bovine serum albumin blocking buffer and incubated at 4 °C overnight with specific primary antibodies against phospho-PERK, phospho-IREα, GRP78/BiP, CHOP (Cell Signaling Technology, Danvers, MA, USA), sorcin, and FliI (Abcam, Cambridge, MA, USA). Signals were detected using an ECL detection kit (GE Healthcare, Chicago, IL, USA) and subsequently incubated with horseradish peroxidase-conjugated secondary antibodies (Thermo Fisher Scientific, Waltham, MA, USA).

### Gene expression analysis

Total RNA was isolated from cells or tissues using TRIzol reagent (Invitrogen). RNA was reverse transcribed using the ABI Reverse Transcription kit. Quantitative PCR was performed with SYBR green fluorescent dye on an ABI 9300 PCR instrument. Relative mRNA expression was determined by the ΔΔCt method and normalized against *TBP* mRNA expression.

### Colon cancer models

Colon tissues from *APC*^min+^- and colitis-induced mouse models of CRC were prepared as previously described^27,28^. Briefly, for colitis-induced CRC, 7- to 8-week-old male mice were intraperitoneally injected with azoxymethane (AOM, 10 mg/kg body weight) and maintained on a regular diet and water for 7 days. Mice were then subjected to five cycles of dextran sodium sulfate (DSS) treatment, in which each cycle consisted of 1.5% DSS for 7 days followed by a 7-day recovery period with regular water.

### Xenograft tumor models

All experiments involving animals were approved by UNIST (IACUC-12-003-A). shRNA-Ctrl or shRNA-FliI CT26 cells (1 × 10^6^) suspended in sterile PBS (200 μl) were injected subcutaneously into the left flanks of 6-week-old BALB/c *nu/nu* male mice (SLC Inc., Hamamatsu, Shizuoka, Japan). Tumor sizes were measured using calipers every 2 days for 17 days. Tumor volume was calculated using the following formula: *V* = 1/2 × (width)^2^ × length. At the end of the experiment, the animals were euthanized, and tumors were collected for western blotting.

### Statistical analysis

Data are presented as the mean ± standard errors of the mean as indicated in the figure legends. Comparisons between two groups were made by unpaired two-tailed Student’s *t* tests. *p* values < 0.05 were considered statistically significant. Microsoft Excel was used for statistical calculations.

## Results

### FliI is upregulated in CRCs

**Fig. 1:**
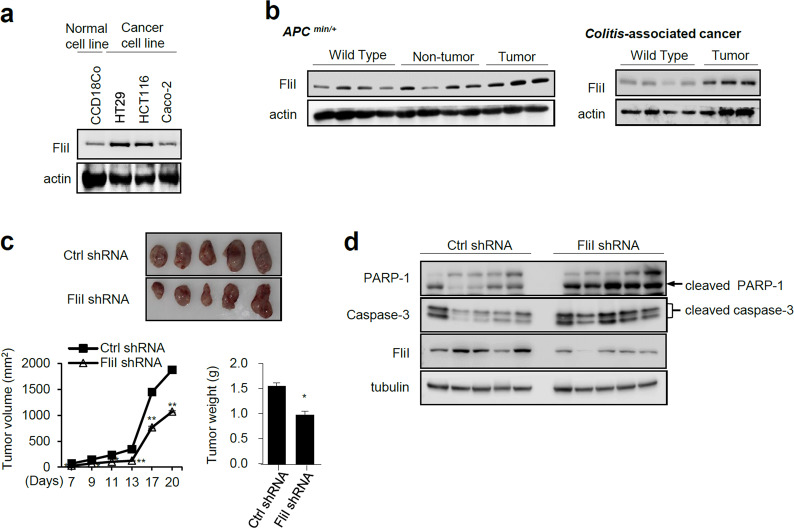
FliI is overexpressed in colorectal cancer. FliI expression in a colon cancer cell line (**a**) and colon tissue from *APC*^min/+^ and colitis-associated cancer mouse models (**b**) were analyzed by western blotting. **c** Orthotopic xenografts of Ctrl or FliI-knockdown CT26 cells were detected (*n* = 5). Images show tumors dissected from the mice. Tumor volume was measured every 2 days, and tumor weight was measured after resection at the end of the experiment. Data are shown as the means ± SEM. *p < 0.05 compared with shRNA-Ctrl cells. **d** Protein levels of cleaved PARP-1, cleaved caspase-3, and FliI were detected in mouse tumor tissue derived by western blotting.

To investigate the clinical relevance of FliI in CRC, we first examined FliI expression in both CRC cell lines and cancer tissue in the *APC*^min+^- and colitis-induced mouse model of CRC. FliI expression in CRC cell lines and cancer tissues was significantly higher than that in the corresponding normal cell line and nontumor tissues (Fig. 1a, b). To directly assess the cell-autonomous function of FliI in mouse CRC, CT26 cells stably transduced with either shRNA-Ctrl or shRNA-FliI lentiviral shRNAs were implanted subcutaneously into immunodeficient mice to form xenograft tumors. In accordance with the results shown in Fig. 1b, knockdown (KD) of FliI significantly repressed xenograft tumor growth (Fig. 1c). Moreover, FliI-KD tumors had elevated levels of cleaved PARP-1 and caspase-3 (Fig. 1d), suggesting that the reduced level of FliI promoted apoptosis in CT26 cells.

### FliI inhibits ER stress-induced apoptosis in CT26 cells

**Fig. 2:**
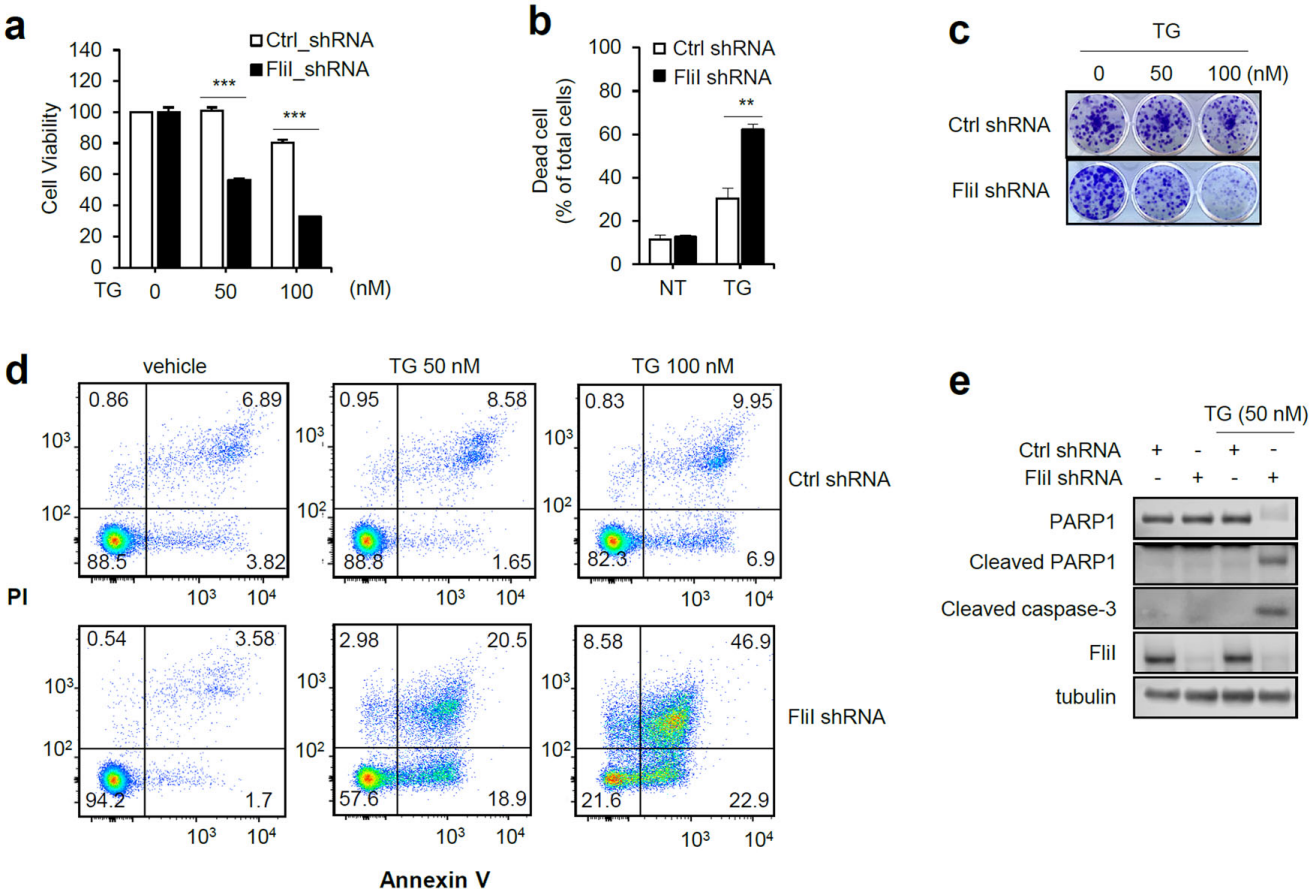
Knockdown of FliI promotes TG-induced apoptosis in CT26 cells. Cell viability of FliI-KD CT26 cells was measured by MTT assay (**a**), cell counting assay (**b**), and colony assay (**c**) after treatment with TG for 48 h. Data are shown as the mean ± SEM. **p < 0.01; ***p < 0.001 shRNA-Ctrl cells vs. shRNA-FliI cells. After Ctrl- and FliI-KD cells were treated with TG for 48 h, they were subjected to annexin V-PI flow cytometry assay (**d**) and western blotting for PARP-1, cleaved caspase-3, FliI, and tubulin (**e**). TG thapsigargin.

The microenvironment within a solid tumor differs from that of normal tissues; the former is characterized by glucose deprivation, low pH, hypoxia, and an imbalance between production and removal of reactive oxidative stress^29^. All of these environmental factors contribute to ER stress; therefore, cancer cells must find effective ways to adapt and prevent ER stress-induced apoptosis^3^. To elucidate the molecular mechanisms by which FliI-KD promotes apoptosis in the xenograft mouse model, we first determined the effect of FliI on cell survival after treatment with the ER stress-inducing drug TG. Cell viability was assessed by MTT and cell counting assays, which revealed that depletion of FliI decreased cell survival following TG treatment (Fig. 2a, b). Colony forming assays confirmed that FliI was required for cell survival following TG treatment (Fig. 2c). Furthermore, FACS analysis using annexin V-PI labeling revealed that depletion of FliI increased ER stress-induced apoptosis (Fig. 2d). Consistent with the FACS analysis, depletion of FliI increased the levels of cleaved PARP-1 and caspase-3 following TG treatment (Fig. 2e). Another ER stress-inducing drug, tunicamycin (TM), significantly increased apoptosis in FliI-KD cells (Supplementary Fig. 1). Together, these data indicate that FliI inhibits ER stress-induced apoptosis.

### Knockdown of FliI sensitizes cells to ER stress

**Fig. 3:**
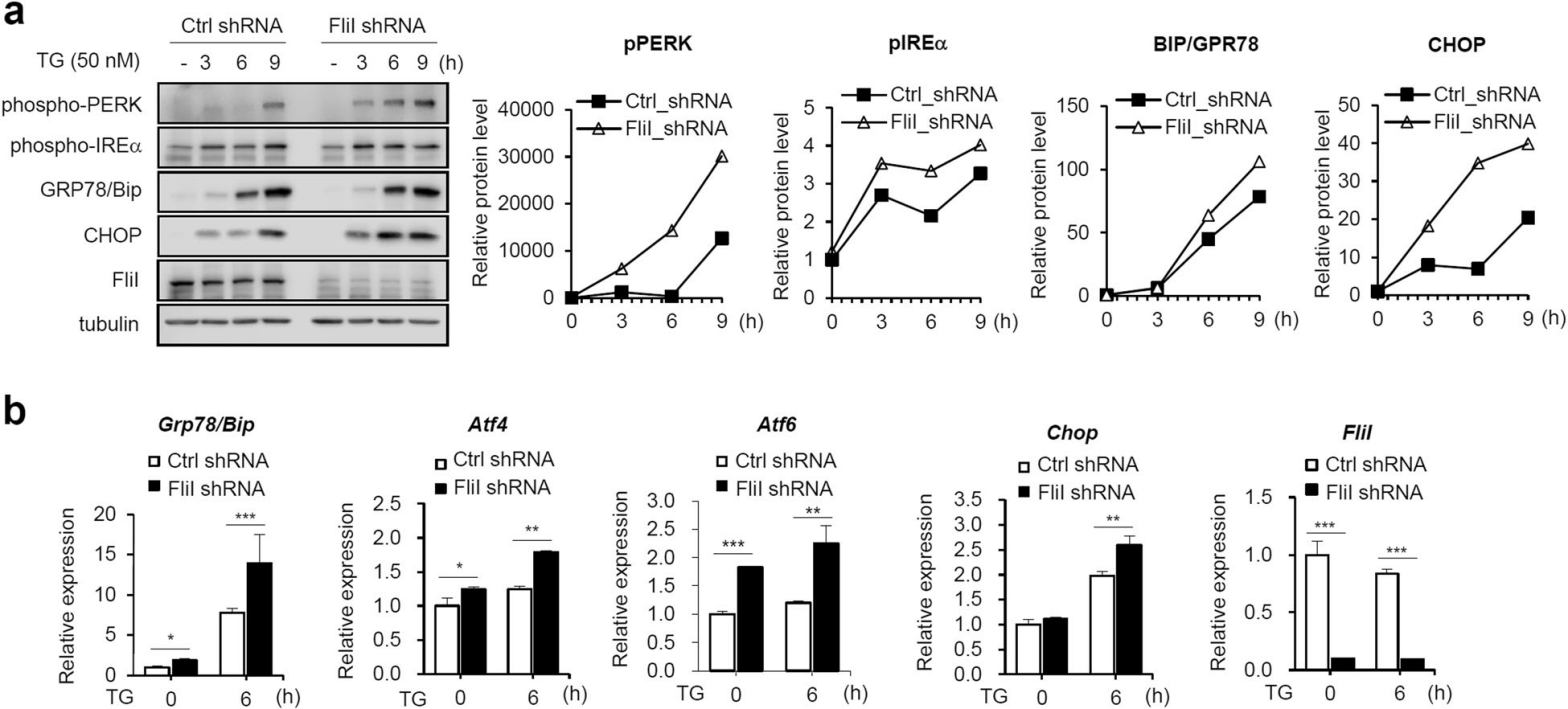
Knockdown of FliI sensitizes CT26 cells to TG-induced UPR. Ctrl- and FliI-KD cells were treated with 50 nM TG for the indicated times, and the extracts were analyzed by western blotting with antibodies against phospho-PERK, phospho-IREα, GRP78/BiP, CHOP, FliI, and tubulin (**a**). mRNA expression was analyzed by quantitative real-time PCR (**b**). Data are shown as the mean ± SEM (*n* = 3). *p < 0.05; **p < 0.01; ***p < 0.001, shRNA-Ctrl cells vs. shRNA-FliI cells. TG thapsigargin.

Disturbances in normal ER functions lead to accumulation and aggregation of unfolded proteins, which initiates an adaptive response, the UPR, to restore normal ER function^6^. Failure to activate the adaptive response results in apoptosis^30^. To obtain insights into the molecular mechanism of action of FliI in ER stress-induced apoptosis, we first evaluated the effect of FliI on the ER stress response. As shown in Fig. 3a, within 3 h of TG treatment, phosphorylation of PERK and IREα was triggered more strongly in FliI-KD cells than Ctrl cells. In addition to UPR sensor protein activation, GRP78/BiP and CHOP levels were also elevated in FliI-KD cells. Similar results were observed after treatment of FliI-KD cells with TM (Supplementary Fig. 2a). Furthermore, the expression of UPR genes, including GRP78/BiP, ATF4, ATF6, and CHOP, was significantly elevated in FliI-KD cells after treatment with TG or TM (Fig. 3b and Supplementary Fig. 2b). These results indicate that KD of FliI made CT26 cells more sensitive to ER stress.

### Knockdown of FliI promotes ER stress-induced apoptosis by disrupting intracellular Ca^2+^ homeostasis

**Fig. 4:**
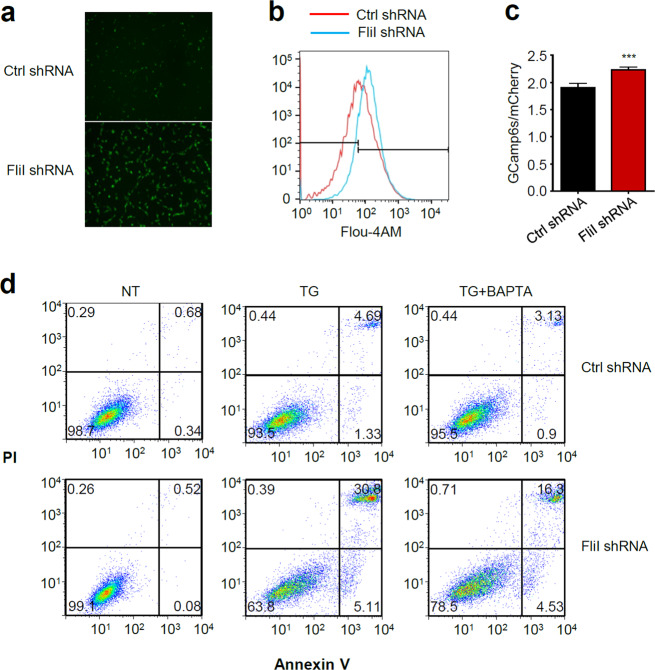
Knockdown of FliI increases the intracellular Ca^2+^ concentration in CT26 cells. Ctrl- and FliI-KD cells were incubated with Fluo-4AM for 30 min, and intracellular Ca^2+^ was (**a**) imaged on a fluorescence microscope (200×) and (**b**) measured by FACS analysis. **c** Ctrl- and FliI-KD cells were transfected with mCherry-GCaMP6s and imaged on a fluorescence microscope. Intracellular Ca^2+^ levels were monitored based on the ratio of GCaMP6s to mCherry fluorescence intensity. **d** Ctrl- and FliI-KD cells were pretreated with BAPTA (10 μM) and treated with TG (100 nM) for 48 h, and then apoptosis was detected by annexin V-PI flow cytometry assay. TG thapsigargin.

Ca^2+^ is a major player in the regulation of apoptosis, both at the early and late stages^31^. Disruption of intracellular Ca^2+^ homeostasis can induce ER stress-mediated apoptosis in response to various pathological conditions^32–34^. Therefore, we next investigated whether ER stress-induced apoptosis sensitized by KD of FliI influence the intracellular Ca^2+^ concentration. Using Fluo-4AM, a calcium-sensing dye, we observed that depletion of FliI triggered upregulation of intracellular Ca^2+^ (Fig. 4a, b). Similarly, using GCaMP6s, the Ca^2+^ indicator, we found that intracellular Ca^2+^ was significantly increased in FliI-KD cells compared with Ctrl cells (Fig. 4c). Furthermore, 1,2-bis(o-aminophenoxy)ethane-N,N,-N′,N′-tetraacetic acid-acetoxymethyl ester (BAPTA-AM), a highly selective Ca^2+^ chelator, abrogated TG-induced apoptosis in FliI-KD CT26 cells (Fig. 4d). These results strongly suggest that elevation of intracellular Ca^2+^ decreases the cellular threshold for TG-induced apoptosis in FliI-KD cells.

### Knockdown of FliI promotes ER Ca^2+^ release through RyR and alteration of intracellular Ca^2+^, resulting in cell death

**Fig. 5:**
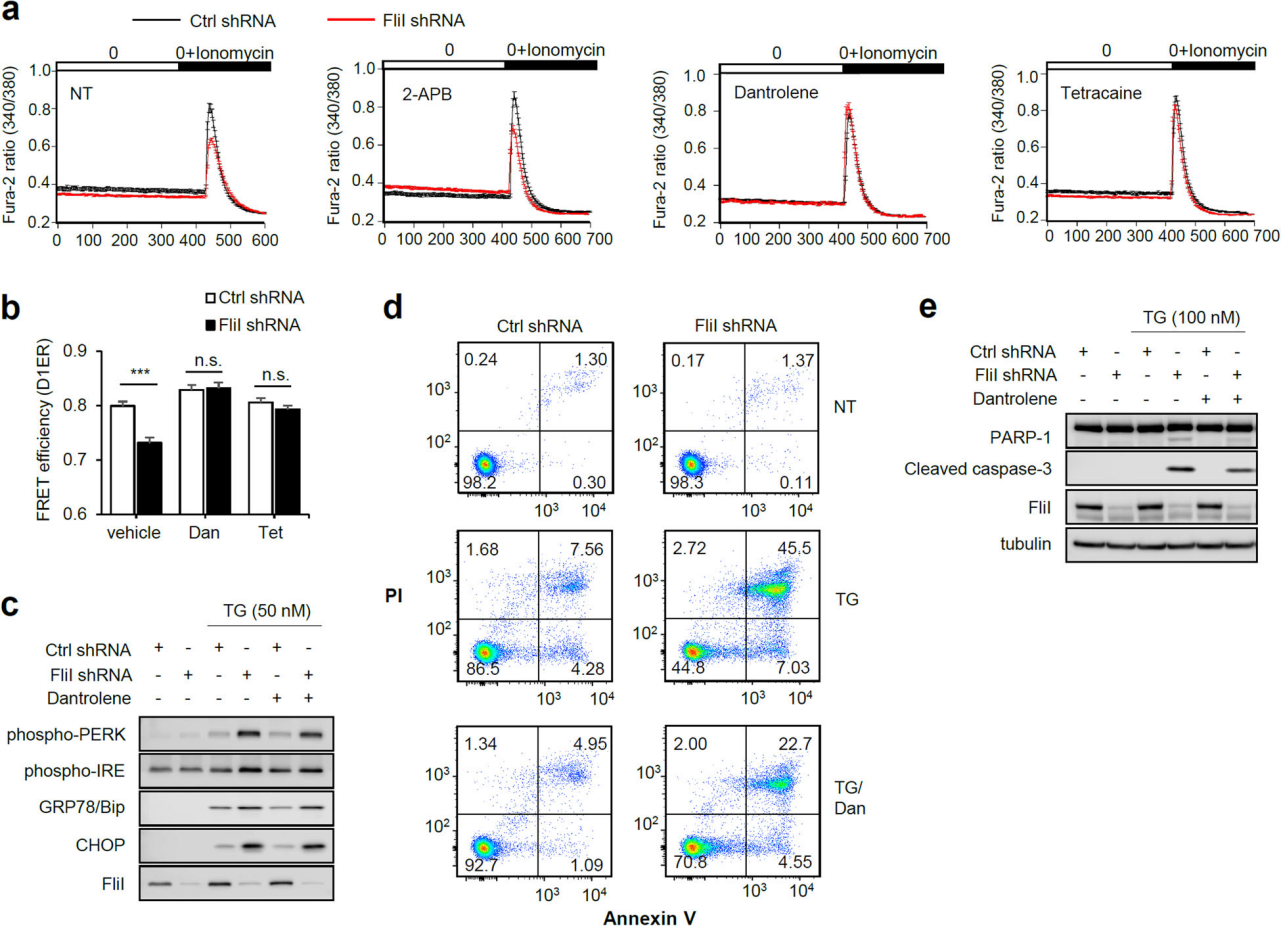
Knockdown of FliI causes ER Ca^2+^ release through RyRs, which induces ER stress-mediated apoptosis in CT26 cells. **a** Ctrl- and FliI-KD cells were pretreated for 2 h with 2-APB (50 μM) and dantrolene (50 μM) and tetracaine (50 μM) and then incubated in Fura-2AM for 30 min. Ratiometric Ca^2+^ imaging was performed in 0 mM Ca^2+^ Tyrode’s solution with or without 5 μM ionomycin, and Ca^2+^ influx was monitored based on the Fura-2 fluorescence ratio. **b** After 24 h transfection of ctrl- and FliI-KD cells with pcDNA-D1ER, dantrolene (50 μM) and tetracaine (50 μM) were treated for 2 h, and the FRET signal was measured. Data are shown as the mean ± SEM. ***p < 0.001, shRNA-Ctrl cells vs. shRNA-FliI cells. Dan dantrolene, Tet tetracaine. **c** Ctrl- and FliI-KD cells were treated with 50 nM TG for 6 h following pretreatment with 50 μM dantrolene. Cell lysates were analyzed by western blotting for phospho-PERK, phospho-IREα, GRP78/BiP, CHOP, FliI and tubulin. **d**, **e** Ctrl- and FliI-KD cells were pretreated for 2 h with dantrolene (20 μM) and then with TG (100 nM) for 48 h. Apoptosis was detected by annexin V-PI flow cytometry assay (**d**) and western blotting for PARP-1, cleaved caspase-3, FliI, and tubulin (**e**). TG thapsigargin, Dan dantrolene.

The ER is the primary site of intracellular Ca^2+^ storage, and ER Ca2^+^ depletion is the major cause of both ER stress and ER stress-induced apoptosis^35^. Hence, we postulated that the ER contributes to the increase in intracellular Ca^2+^ caused by FliI-KD. To test this idea, we determined the concentration of free Ca^2+^ within the ER by depleting the ER Ca^2+^ store with the ionophore ionomycin. As shown in Fig. 5a, ionomycin treatment increased intracellular Ca^2+^, and the ionomycin-mediated intracellular Ca^2+^ increase was lower in FliI-KD cells than in control cells. In addition, we measured cytosolic Ca^2+^ released from the ER by treatment with TG or DBHQ, a specific inhibitor of SERCA, to deplete ER Ca^2+^. Consistent with the results in Fig. 5a, the intracellular Ca^2+^ concentration was lower in TG- or DBHQ-treated FliI-KD cells (Supplementary Fig. 3a, b). Furthermore, when we exposed FliI-KD cells to a low dose (1 μM) of DBHQ for short (30 min) and long periods (3 h), ER Ca^2+^ depletion of FliI-KD was observed to occur in a time-dependent manner (Supplementary Fig. 3c), clearly indicating that the elevation in intracellular Ca^2+^ in FliI-KD cells was caused by the release of ER Ca^2+^. Finally, we directly measured the ER Ca^2+^ concentration using an ER-targeted Cameleon (D1ER) probe. As expected, ER Ca^2+^ was lower in FliI-KD cells than in control cells (Fig. 5b).

Regulation of intracellular Ca^2+^ by ER is mainly mediated by Ca^2+^ reuptake into the ER through SERCA Ca^2+^ pumps and Ca^2+^ release through Ca^2+^ channels, including IP_3_Rs or RyRs^8–10^. Therefore, we next investigated whether the increase in intracellular Ca^2+^ in FliI-KD cells was mediated by ER Ca^2+^ channels. For these experiments, we used two different Ca^2+^ channel blockers, dantrolene, tetracaine, and 2-aminoethoxydiphenyl borate (2-APB), which are inhibitors of RyRs and IP_3_Rs, respectively. Dantrolene and tetracaine elevated the ionomycin-induced increase in intracellular Ca^2+^ in FliI-KD cells, whereas 2-APB did not (Fig. 5a). Dantrolene and tetracaine also elevated TG-induced and DBHQ-induced intracellular Ca^2+^ increases in FliI-KD cells (Supplementary Fig. 3a, b). Consistent with the results of intracellular Ca^2+^, ER Ca^2+^ was also recovered by treatment with dantrolene and tetracaine in FliI-KD cells (Fig. 5b). Furthermore, dantrolene and tetracaine inhibited TG-induced expression of ER stress marker proteins (Fig. 5c and Supplementary Fig. 4a). We then tested whether RyR-mediated Ca^2+^ release in FliI-KD cells was associated with TG-induced apoptosis. As shown in Fig. 5d and Supplementary Fig. 4b, inhibition of RyR compromised the increase in TG-induced apoptosis caused by FliI-KD. In addition, the TG-induced increases in the levels of cleaved PARP-1 and caspase-3 in FliI-KD cells were rescued by dantrolene treatment (Fig. 5e). Together, these results strongly suggest that RyR channel-dependent ER Ca^2+^ release is an essential upstream event in the sensitization of FliI-KD cells to TG-induced apoptosis.

### Knockdown of FliI suppresses the expression of sorcin, which protects against ER stress

**Fig. 6:**
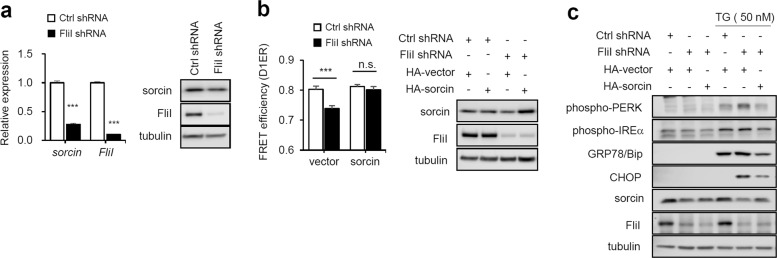
FliI regulates ER Ca^2+^ through sorcin. **a** Sorcin expression levels were analyzed by western blot and quantitative real-time PCR. Data are shown as the mean ± SEM (*n* = 3). ***p < 0.001, shRNA-Ctrl cells vs. shRNA-FliI cells. **b** After cotransfection of vector or sorcin with D1ER, FRET signals were measured. Data are shown as the mean ± SEM. ***p < 0.001, shRNA-Ctrl cells vs. shRNA-FliI cells. **c** Ctrl- and FliI-KD cells were treated with 50 nM TG for 6 h following expression of vector or sorcin. Cell lysates were analyzed by western blotting for phospho-PERK, phospho-IREα, GRP78/BiP, CHOP, sorcin, FliI, and tubulin.

To investigate the molecular mechanism by which FliI regulates Ca^2+^ release through RyRs, we compared the global gene expression profile using RNA sequencing. We focused on sorcin (soluble resistance-related calcium-binding protein) downregulation by FliI-KD (Fig. 6a) because sorcin has been reported to negatively regulate ER Ca^2+^ release by mediating RyR and SERCA^36,37^. Then, we hypothesized that downregulated sorcin contributes to the depletion of ER Ca^2+^ through RyR in FliI-KD cells. To verify this speculation, we overexpressed sorcin in FliI-KD cells and measured ER Ca^2+^. As shown in Fig. 6b, decreased ER Ca^2+^ was recovered by overexpression of sorcin in FliI-KD cells. Furthermore, sorcin overexpression in FliI-KD cells decreased the level of TG-induced ER stress compared with that in vector-expressed FliI-KD cells (Fig. 6c). These results suggest that FliI controls the ER Ca^2+^ pool by regulating sorcin expression.

## Discussion

Globally, CRC is the third most commonly diagnosed malignancy and the second leading cause of cancer-related death^1^. In particular, the emergence of refractory disease following chemotherapy continues to be a major contributor to treatment failure and to the high mortality rates observed in advanced CRC. We now know that the outcome of anticancer treatment can be influenced by the cancer cell response to cellular stress induced by chemotherapeutic agents. The ER stress response is an adaptive mechanism that protects the cell against environmental stimuli^4^. In addition, the ER has been implicated in promoting apoptosis by releasing calcium, which serves as a secondary messenger for mitochondrion-mediated apoptosis^38^. In a tumor context, high proliferation rates and accelerated metabolism burden cancer cells with cellular and metabolic stress; therefore, as an adaptation, these cells generally activate the ER stress response to support their own survival^39^. However, this signaling event is a double-edged sword: although it supports cancer cell survival in an adverse environment, harsh and sustained stress conditions, such as those induced by chemopreventive agents, tip the balance from prosurvival to proapoptosis, culminating in ER stress-mediated cancer cell death^39^. The mechanisms determining the switch from adaptation to cell death signaling under ER stress have not been clearly defined, and the outcome of ER stress signaling may depend on the type, intensity, and duration of the stimulus in relation to active changes in the cellular environment. Despite this lack of clarity, employing strategies to either sensitize or persuade the cancer cells to ER stress may prove to be a useful tool for overcoming drug resistance and proliferation in cancer cells. Notably, van den Brink et al. reported that ER stress-induced differentiation sensitizes colon cancer stem cells to chemotherapy^40^. Many studies have supported the idea that FliI plays important roles in tumor progression by stimulating cell proliferation, inhibiting apoptosis, and promoting invasion^21–23,41^. In this study, we found that FliI is highly expressed in CRC and that KD of FliI in CT26 cells promoted the induction of ER stress by TG, resulting in apoptosis (Figs. 2, 3). The molecular mechanism by which FliI influences sensitivity to ER stress inducers remains unclear, but we speculate that one important factor is the Ca^2+^ concentration in the ER and cytosol.

Calcium, stored primarily in the ER, is one of the key regulators of cell survival, but it can also induce apoptosis in response to a variety of pathological conditions^29^. Furthermore, calcium-mediated signaling pathways were implicated in tumorigenesis and tumor progression, including metastasis, invasion, and angiogenesis^42,43^. Conversely, several studies showed that sustained intracellular calcium overload can trigger cell death or deregulate potential calcium-dependent tumorigenic pathways^29^. Recent studies suggest that interfering with the sequestration of calcium into intracellular pools from the ER can trigger apoptosis as part of a stress response^11,38^. Thus, the final outcome is determined by the amplitude of the increase and the duration of the change in intracellular calcium level, as well as the nature of the change and the location. In this study, downregulation of FliI in CT26 cells increased intracellular Ca^2+^ but decreased ER Ca^2+^ concentration. This Ca^2+^ depletion was mediated by RyRs (Fig. 5). Thus, depletion of FliI resulted in an increase in the intracellular Ca^2+^ concentration through Ca^2+^ release via RyRs, which sensitized cells to ER stress inducers. Thereafter, more rapid and marked increases in intracellular Ca^2+^ levels triggered apoptosis.

We next asked how FliI regulates Ca^2+^ release through RyRs. RyRs are modulated directly or indirectly by Ca_V_1.1/1.2, various ions, and small molecules and proteins, including Ca^2+^, Mg^2+^, protein kinase A, FK506 binding proteins, calmodulin, Ca^2+^/calmodulin-dependent protein kinase II (CaMKII), calsequestrin, and triadin^37^. Most RyR modulators interact with the cytoplasmic region of the channel. Hence, we first hypothesized that FliI stimulates RyR activation because FliI regulates signaling pathways by interacting with signaling proteins^12,13^. FliI interacts directly with active CaMKII to inhibit its signaling cascade^44^, and CaMKII phosphorylates RyRs in the cytosol, altering their gating properties, leading to Ca^2+^ release from the ER^45^. Based on these observations, we postulated that FliI might block the phosphorylation of RyRs by direct interaction with CaMKII, which inhibits RyR activation. However, we did not observe any change in TG-induced UPR signaling or apoptosis when we treated cells with KN-93, a CaMKII inhibitor (data not shown). Therefore, CaMKII activation is not involved in RyR activation by FliI depletion.

Focusing on FliI as a coactivator of transcription factors, we next hypothesized that FliI regulates RyR activation by modulating the expression of genes related to RyRs. Because there was no change in RyR expression in FliI-KD cells (data not shown), we compared global gene expression profiles using RNA sequencing and observed that sorcin (soluble resistance-related calcium-binding protein) was downregulated by depletion of FliI (Fig. 6a). Sorcin is a member of the penta-ERF-hand protein family that localizes to the cytosol, nucleus, plasma membrane, cytoplasmic vesicles, and ER membrane^36,44^. In cardiomyocytes, sorcin interacts with RyRs and SERCA; through these interactions, Ca^2+^-bound sorcin negatively regulates the release of Ca^2+^ from the ER and increases the Ca^2+^ load in the ER by inhibiting RyRs and activating SERCA^36,37^. These changes promote the accumulation of Ca^2+^ in the ER and decrease the level of intracellular Ca^2+^, thereby preventing ER stress. As expected, overexpression of sorcin in FliI-KD cells increased the ER Ca^2+^ pool, which recovered TG-induced ER stress (Fig. 6). Sorcin is upregulated under ER stress^46^ and overexpressed in many human tumors, including leukemia, gastric, breast, and ovarian cancers^47–50^, and in chemoresistant cell lines^51–55^. Notably, in this regard, overexpression of sorcin is associated with multidrug resistance (MDR), whereas downregulation of the protein increases sensitivity to antitumor drugs^56,57^. Moreover, sorcin overexpression is correlated with upregulation of *MDR1/P-glycoprotein*^54,57^, and the P-glycoprotein-dependent MDR phenotype seems to be related to intracellular Ca^2+^ homeostasis^58^. In fact, we also observed downregulation of *MDR1/P-glycoprotein* and ABCB1 amplicons, including *Dbf4*, *Crot*, and *Slc5a40*, whose overexpression confers MDR (Supplementary Fig. 5)^59^. Thus, it is intriguing to speculate that sorcin is involved in FliI-mediated regulation of Ca^2+^ homeostasis, possibly by activating RyRs, which may be responsible for sensitizing cells to ER stress inducer-mediated apoptosis. Further studies are needed to show how FliI regulates sorcin expression.

In conclusion, we have uncovered a novel molecular mechanism in which depletion of FliI increases intracellular Ca^2+^ concentration in CRC cells by promoting ER Ca^2+^ release through RyRs, which sensitizes the cells to ER stress inducers and promotes ER stress. Ultimately, this results in ER stress-induced apoptosis. This study provides the first evidence that FliI plays a role in ER stress by controlling Ca^2+^ homeostasis in CRC cells. In addition, FliI expression is correlated with the expression of sorcin as well as *ABCB1* and its amplicons, which are markers of MDR in CT26 cells. Therefore, targeting FliI represents an innovative and effective strategy for the development of cancer therapeutics.

## Supplementary information

Supplementary Figure
