# Supplementary material for: Flightless-1 inhibits ER stress-induced apoptosis in colorectal cancer cells by regulating Ca2+ homeostasis
Source: Exp Mol Med. 2020 Jun 5;52(6):940–50. doi: 10.1038/s12276-020-0448-3 (PMC7338537; doi:10.1038/s12276-020-0448-3)
Supplement: Supplementary file 1 — Supplementary Figure [file 12276_2020_448_MOESM1_ESM.pdf]

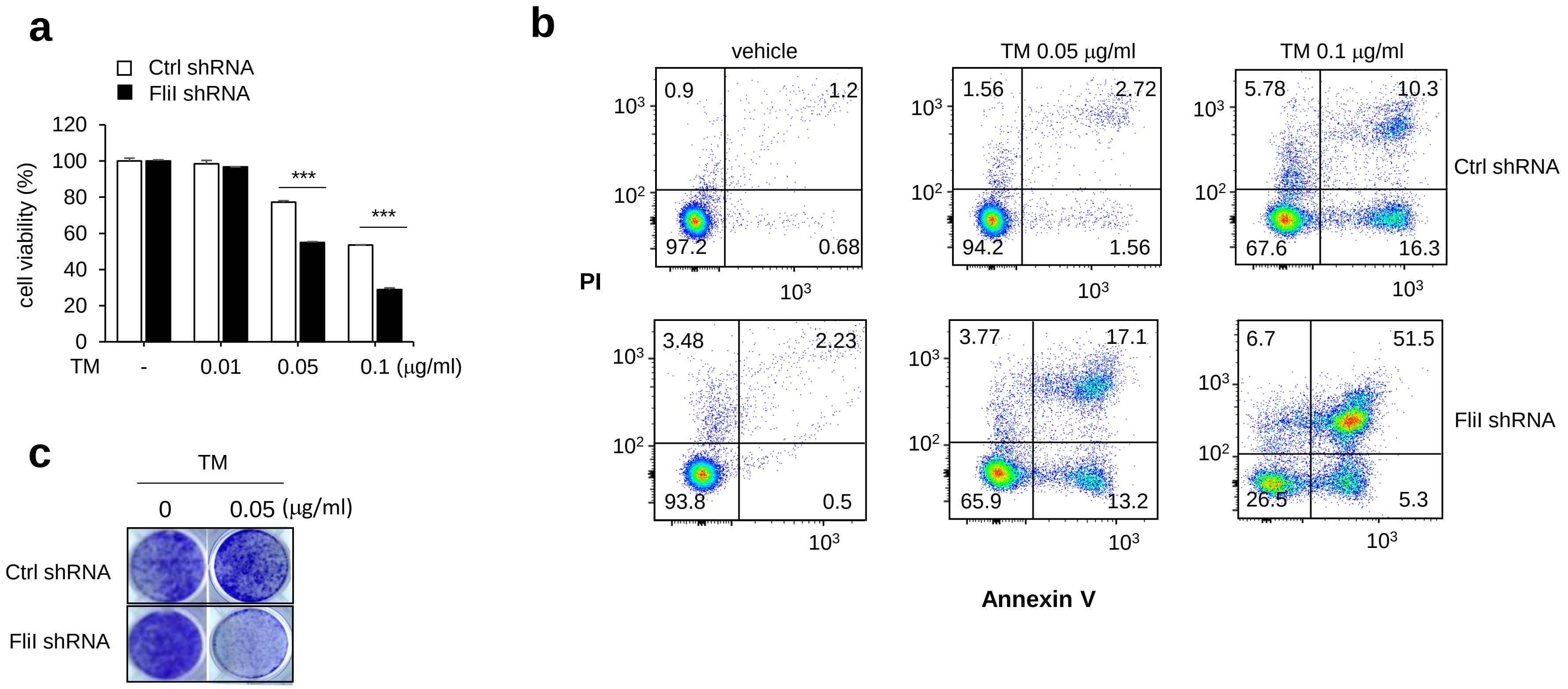

Supplemental Figure 1. Knockdown of FliI promotes TM-induced apoptosis in CT26 cells. Cell viability of FliI-KD CT26 cells was measured by MTT assay (a) and colony assay (c) after treatment with TM for 48 h. Data are shown as means ± S.E.M. $^{***}p < 0.001$ *vs.* shRNA-Ctrl cells. (b) Ctrl- and FliI-KD cells were treated with TM for 48 h and then subjected to annexin V-PI flow cytometry assay. TM, tunicamycin

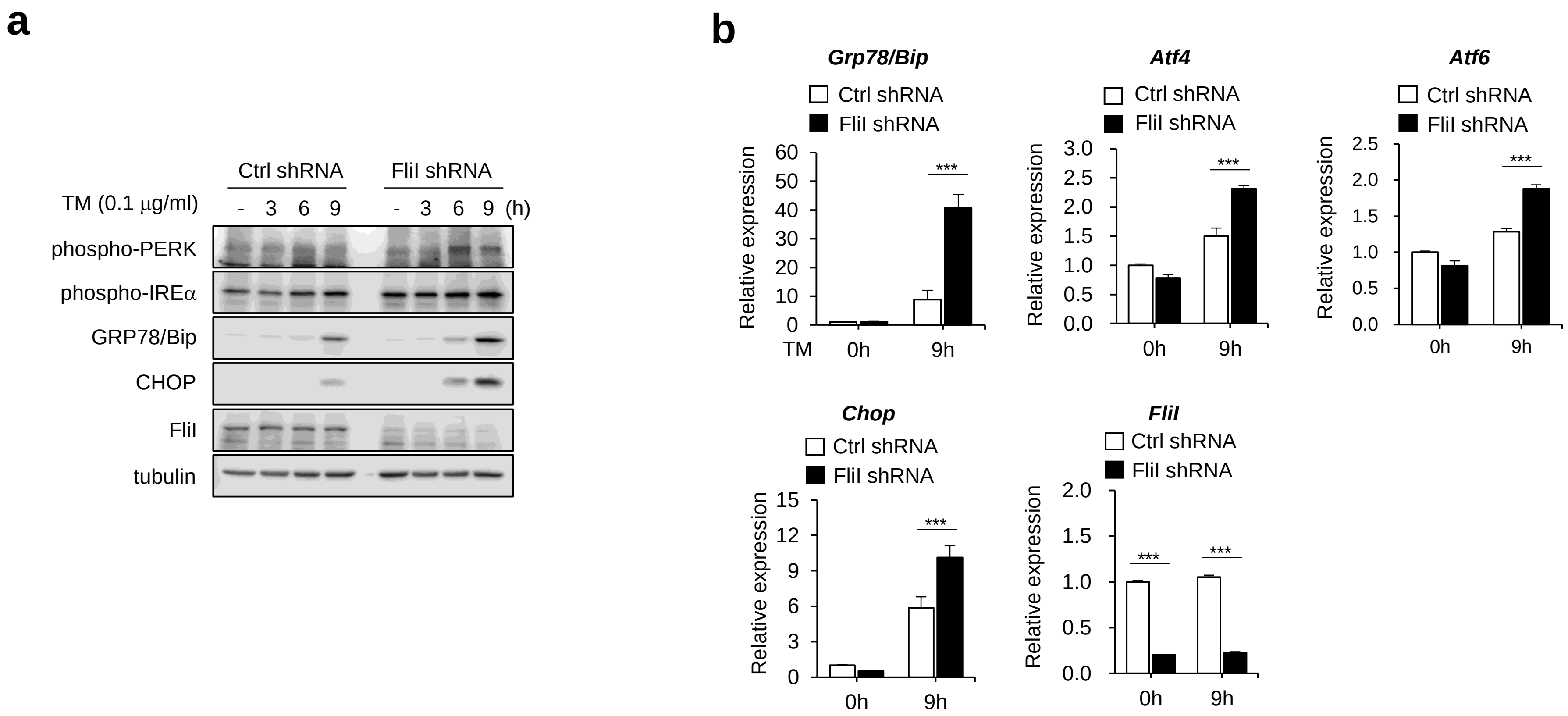

Supplemental Figure 2. Knockdown of FliI sensitizes TM-induced UPR in CT26 cells. Ctrl- and FliI-KD cells were treated with 0.1 μg/ml TM for the indicated times, and extracts were analyzed by western blotting for phospho-PERK, phospho-IREα, GRP78/BiP, CHOP, FliI, and tubulin blot (a). mRNA expression was analyzed by quantitative real-time PCR (b). Data are shown as means ± S.E.M. (*n* = 3). $^{***}p < 0.001$ *vs.* shRNA-Ctrl cells. TM, tunicamycin

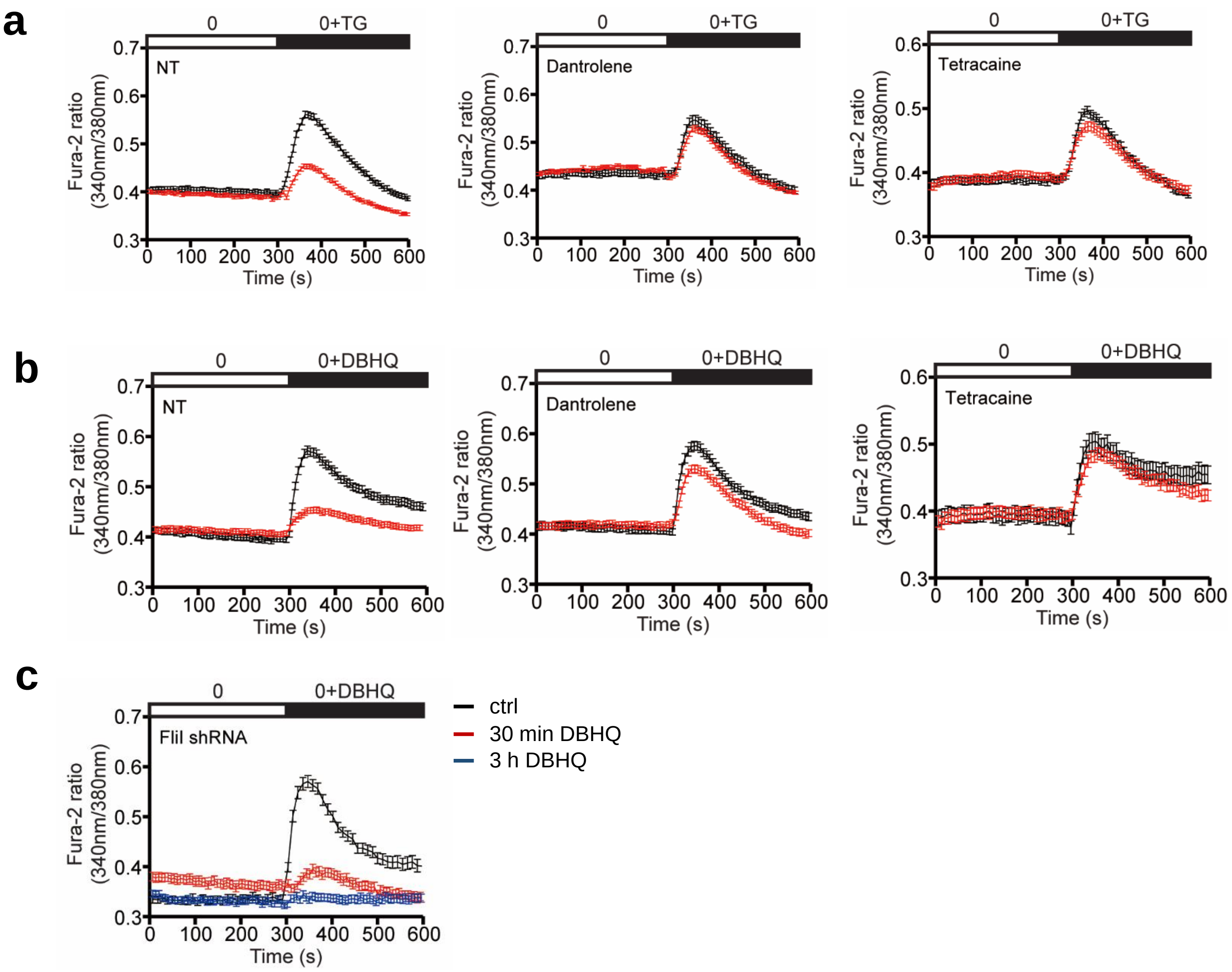

Supplemental Figure 3. Knockdown of FliI causes ER Ca^{2+} release through RyRs in CT26 cells. Ctrl- and FliI-KD cells were pre-treated for 2 hours with dantrolene (50 μM) and tetracaine (50 μM) and then incubated in Fura-2AM for 30 min. Ratiometric Ca^{2+} imaging was performed in 0 mM Ca^{2+} Tyrode's solution with or without 1 μM thapsigargin (a) or 50 μM DBHQ (b) and Ca^{2+} influx was monitored based on the Fura-2 fluorescence ratio. (c) FliI-KD cells were pre-treated for 0, 30 min, 3 hours with DBHQ (1 μM) and then incubated in Fura-2AM for 30 min. Ratiometric Ca^{2+} imaging was performed in 0 mM Ca^{2+} Tyrode's solution with or without 50 μM DBHQ and Ca^{2+} influx was monitored based on the Fura-2 fluorescence ratio. TG, thapsigargin; DBHQ, 2,5-Di-tert-butylhydroquinone.

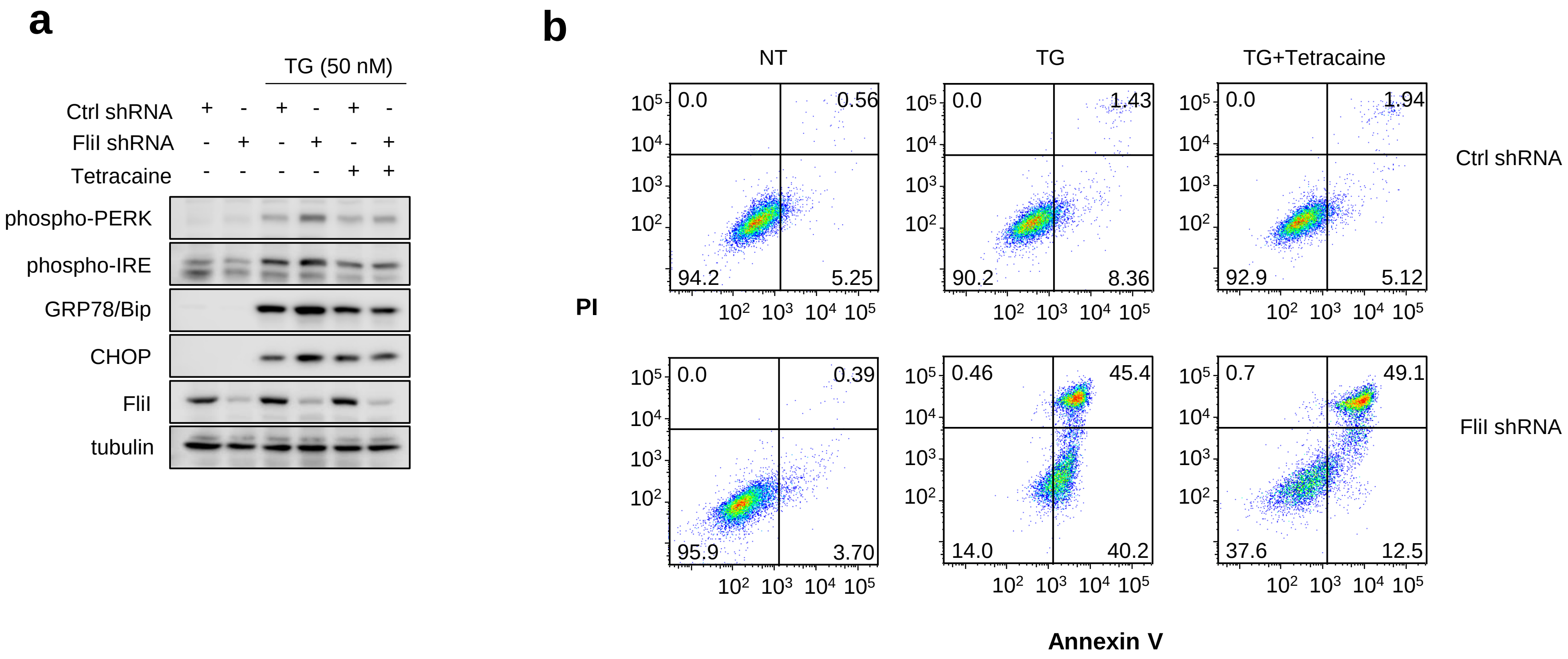

Supplemental Figure 4. RyR-mediated ER Ca^{2+} release induces ER stress-induced apoptosis in FliI KD-CT26 cells. (a) Ctrl- and FliI-KD cells were treated with 50 nM TG for 6 hours following pre-treatment with 50 μM tetracaine. Cell lysates were analyzed by western blotting for phospho-PERK, phospho-IREα, GRP78/BiP, CHOP, FliI and tubulin. (b) Ctrl- and FliI-KD cells were pre-treated for 2 hours with tetracaine (20 μM) and then with TG (50 nM) for 48 hours. Apoptosis was detected by annexin V-PI flow cytometry assay. TG, thapsigargin

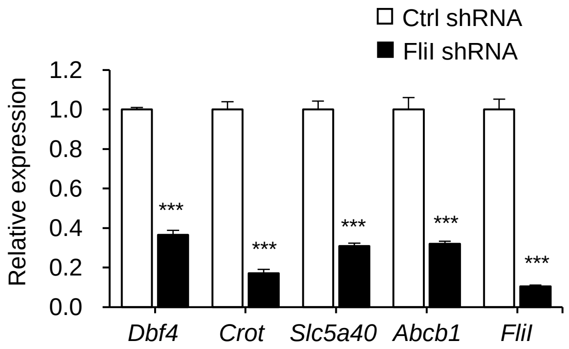

Supplemental Figure 5. Knockdown of FliI decreases MDR-related gene expression in CT26 cells. MDR-related gene expression was analyzed by quantitative real-time PCR. All data are shown as means ± S.E.M. (n = 3). $^{***}p < 0.001$ *vs.* shRNA-Ctrl cells.
